# Parsing heterogeneity in global and local white matter integrity at different stages across the psychosis continuum

**DOI:** 10.1038/s41537-024-00516-7

**Published:** 2024-11-13

**Authors:** Galya C. Iseli, Sarah Ulrich, Philipp Stämpfli, Erich Studerus, David Coynel, Anita Riecher-Rössler, Philipp Homan, Stefan Kaiser, Stefan Borgwardt, Matthias Kirschner, André Schmidt

**Affiliations:** 1 University of Basel, Department of Clinical Research (DKF), University Psychiatric Clinics (UPK), Translational Neurosciences, Basel, Switzerland; 2 Division of Cognitive Neuroscience, Department of Biomedicine, University of Basel, Basel, Switzerland; 3 Experimental Cognitive and Clinical Affective Neuroscience (ECAN) Laboratory, Department of Clinical Research (DKF), Basel, Switzerland; 4 Center for Affective, Stress and Sleep Disorders, University Psychiatric Clinics (UPK), Basel, Switzerland; 5 Department of Adult Psychiatry and Psychotherapy, University of Zurich, Zurich, Switzerland; 6 Neuroscience Center Zurich, University of Zurich and ETH Zurich, Zurich, Switzerland; 7 MR-Center of the Psychiatric Hospital and the Department of Child and Adolescent Psychiatry, University of Zurich, Zurich, Switzerland; 8 Division of Personality and Developmental Psychology, Department of Psychology, University of Basel, Basel, Switzerland; 9 Medical Faculty, University of Basel, Basel, Switzerland; 10 Division of Adult Psychiatry, Department of Psychiatry, Geneva University Hospitals, Geneva, Switzerland; 11 Translational Psychiatry, Department of Psychiatry and Psychotherapy, University of Lübeck, Ratzeburger Allee 160, 23562 Lübeck, Germany

**Keywords:** Psychosis, Schizophrenia, Neuroscience

## Abstract

Psychosis progresses along a continuum. While heterogeneity is evident across the continuum, it remains unknown whether this is also reflected in white matter (WM) heterogeneity and whether parsing WM heterogeneity may reveal subgroups with more pronounced clinical features. This analysis included 212 participants consisting of healthy controls (HC, *n* = 59), individuals with high schizotypy (SPT, *n* = 27), at-risk mental state (ARMS, *n* = 35), and patients with first episode psychosis (FEP, *n* = 50) and schizophrenia (SZ, *n* = 41). Fractional anisotropy (FA) and mean diffusivity (MD) were derived from diffusion tensor imaging (DTI), and fibre density (FD), a non-tensor-derived diffusion marker, was computed. The Person-Based-Similarity Index (PBSI) and Coefficient of Variation Ratio (CVR) were computed to assess global and local heterogeneity. ANOVAs were performed to determine whether people with deviating PBSIs exhibit more pronounced clinical features. Global heterogeneity for all diffusion parameters significantly differed across groups, with greatest difference in heterogeneity between SZ and HC. Results further indicate that FA deviators exhibit lower global functioning and higher negative symptoms. Local FA heterogeneity was greater in FEP relative to ARMS and HC in almost all WM tracts, while SZ patients specifically showed greater heterogeneity in the right thalamic radiation and the left uncinate compared to HCs. Group differences in WM heterogeneity might be indicative of symptom specificity and duration. While these findings offer valuable insights into the neurobiological variability of psychosis, they are primarily hypothesis-generating. Future large-scale studies are warranted to test the robustness of diffusion markers and their clinical relevance.

## Introduction

Acute psychosis is greatly debilitating, and amongst the highest weighted global burdens of disease^1^. Psychosis occurs along a continuum ranging from stages with subclinical psychotic like experiences in healthy subjects from the general population (e.g., people with schizotypy) and individuals at clinical high-risk for psychosis (CHR), not bound to transition^2,3^, and further to patients with first-episode psychosis (FEP) and schizophrenia (SZ)^4^. This classification into stages primarily enabled diagnosis and has advanced our understanding of pathogenesis and aetiology^5^, yet psychosis is regarded as inherently heterogeneous in symptom presentation, functioning and treatment response^6^. Clinical heterogeneity is apparent at each stage across the psychosis continuum^6–8^ and accompanied by heterogeneity in brain structure as well as function^9^. Dissecting brain heterogeneity within and across stages on the continuum may help to identify more homogeneous subgroups with common clinical manifestations and in turn enhance preventive treatment responses and outcomes to more targeted interventions, proportionate to clinical need^10^.

Magnetic resonance imaging (MRI) has become an important non-invasive tool, supporting our understanding of structural brain abnormalities in psychosis and their respective symptom expression^11^, with evidence for whole-brain grey matter (GM) alterations at each stage across the psychosis continuum. However, such mean group comparisons did not establish any reliable neuroanatomical biomarkers, probably because case-control analyses do neglect within-group variability^12,13^. Seminal works addressed this gap by first exploring interindividual variability of regional grey matter volume (GMV) in schizophrenia. Brugger and Howes investigated the variability of regional GMV by utilising the log variability ratio (VR) and log coefficient of variation ratio (CVR) and found significant differences in the variability of regional GM in SZ patients^14^. Later studies, utilising the CVR, consistently reported greater cortical and subcortical volume heterogeneity in SZ patients referenced to controls^15^, yet report no significant regional heterogeneity difference in CHR relative to a healthy control (HC) sample in structural MRI measures of cortical surface are (SA), cortical thickness (CT), and subcortical volume (SV)^16^.

The Person-Based-Similarity Index (PBSI) has recently been introduced as an index of global brain heterogeneity, with the advantage of returning personalised estimates of the degree of similarity of single individuals to the normative reference or within the group^17^. By utilising the PBSI as a personalised metric to investigate inter-subject correlation, a greater within-group variability (lower PBSI scores) in CHR subjects compared to HCs was confirmed for SA, CT, and SV^16^. Antoniades et al.^18^ further showed that relative to HCs, CHR individuals and FEP patients also revealed a lower PBSI for CT, SA and SV. Moreover, they could also show that individuals with deviating PBSI scores had lower IQ and higher psychopathology^18^, indicating the potential of normative models to identify individuals with more prominent clinical manifestations^13^.

Diffusion tensor imaging (DTI) studies demonstrated widespread white matter (WM) microstructural differences in SZ. The largest study so far, conducted in 4322 patients, reported reduced fractional anisotropy (FA) and increased mean diffusivity (MD) across all major WM fasciculi^19^. Previously, we also showed that a new, non-tensor-derived diffusion marker, the measure of fibre density (FD), may be more sensitive to subtle changes in WM microstructure compared to FA in SZ^20^. Such deficits in WM integrity have been reported in early psychosis^21–23^ and related to a number of affective, cognitive, and perceptual symptoms observed in psychosis,^24–26^ as well as to treatment response^27^. Recent works have focused on multimodal variability in patients^28^, however, to the best of our knowledge, no study has yet compared variability in WM integrity among different stages along the psychosis continuum.

In this study, we investigate global (via PBSI) and local (via CVR) heterogeneity differences in FA, MD, and FD among HCs, those with schizotypy and CHR, FEP and patients with SZ, and whether individuals with deviating PBSI score exhibited more pronounced clinical features. In line with previous works in FEP and CHR^16,18^ we predicted lower PBSI for all diffusion metrics in schizotypy, CHR, FEP and SZ compared to HCs and that deviators would express lower IQ and higher psychopathology. Furthermore, for subclinical stages on the psychosis continuum^29^, hence schizotypy and CHR, we further anticipated increased regional heterogeneity compared with HC in the corpus callosum^26,30–33^, bilateral anterior corona radiata, the bilateral thalamic radiation, and the left superior fronto-occipital fasciculus^26^, and expect these effects predominantly in FA due to its suggested early-stage-specificity^26,34,35^. In later stages of clinical progression (i.e. FEP and SZ patients), we expect to see increased heterogeneity in callosal and commissural fibre tracts and fronto-temporal-limbic pathways^36^.

## Methods

This is an ad-hoc multisite analysis of previously conducted site-specific investigations in ARMS^24,37^, SPT^38^, FEP^20,24^, and SZ^20,39^. Here, we report pooled findings on global (using the PBSI) and local heterogeneity (using CVR) in WM integrity across the different stages along the psychosis spectrum.

### Participants

Data of 212 participants collected at three different centres was included. Datasets were collected at the Department of Psychiatry (UPK) of the University of Basel, the University Hospital of Psychiatry, Bern and the Department of Psychiatry, Psychotherapy and Psychosomatics at the Psychiatric Hospital, University of Zurich. All participants provided written and informed consent, and the study was approved by the respective local ethics committees. Samples ranging from five different stages along the psychosis continuum were included in the study: 59 HC, 35 ARMS, 27 SPT, 50 FEP patients, and 42 patients with SZ.

The Basel sample consisting of ARMS (*n* = 35) and FEP (*n* = 36) was recruited in an open prospective clinical study for the Early Detection of Psychosis (FePsy)^40^. ARMS subjects were recruited according to the Personal Assessment and Crisis Evaluation (PACE)^24^ and the “Basel Screening Instrument for Psychosis” (BSIP)^41^, a screening checklist for help seeking populations which is performed by experienced psychiatrists and consists of similar criteria to the Comprehensive Assessment of ARMS questionnaire^42^.

FEP patients from the Basel sample fulfilled the criteria for first-episode psychosis according to the ICD-10 or DSM-IV^43^ but not yet for schizophrenia^37,44^. Inclusion criteria were scores of 4 or above on the hallucination item, of 5 or above on the unusual thought content, suspiciousness or conceptual disorganisation items, from the Brief Psychiatric Rating Scale (BPRS)^37,45^. The symptoms had to have occurred multiple times and persisted for more than one week^37^. At the time of scanning, ten of the included 36 FEP patients from the Basel sample were receiving antipsychotic treatment, five were receiving antidepressant treatment and six receiving both of unknown duration. Twelve FEP patients received neither (missing data for 3 patients).

The SZ patients from Bern (*n* = 21) came from the SWIFT-study and were diagnosed with schizophrenia spectrum disorder according to the DSM-IV-TR criteria^39,46,47^. The diagnoses were confirmed by an experienced clinician and the Mini International Neuropsychiatric Interview (MINI)^39,48^. Further inclusion criteria for patients were age between 18 and 65 years, sufficient fluency in German and right handedness^39,47^. Exclusion criteria entailed left-handedness, pregnancy, history of serious neurological issues, or reported current abuse of alcohol and/or psychoactive substances apart from nicotine^39^.

The Zurich sample consisted of FEP patients (*n* = 14) who were recruited during their first psychiatric admission in outpatient and inpatient units of the Psychiatric Hospital of the University Zurich. The inclusion criterion of a diagnosis of brief psychotic disorder, schizophreniform disorder or schizophrenia was confirmed in a structured Mini-International Neuropsychiatric Interview for DSM-IV (MINI)^38,48^. All FEP patients from the Zurich sample received a stable dose of second-generation antipsychotics^38,49^. Healthy individuals with high SPT (*n* = 27) were recruited using an online form of the Schizotypal Personality Questionnaire (SPQ)^50^. Out of 956 participants who completed the questionnaires, the individuals scoring highest (upper 10% of maximum SPQ score) were included in the study^38,49^. SZ patients (*n* = 20) from the Zurich sample were assessed with the structured MINI in accordance with DSM-IV criteria^20,38,39^. Patients with any other Axis I DCM-IV disorder, such as current substance use disorder or major depression were excluded from the study^20^. All included patients with SZ received a stable dose of second-generation antipsychotic, with no change in medication or dose for at least two weeks and were clinically stable^20^.

HCs were recruited from the same geographical areas as the other groups and data collected across all sites. They had no history of psychiatric illness, head trauma, neurological illness, serious medical or surgical illness, substance abuse, and no family history of any psychiatric disorders, which was assessed in a detailed semi-structured interview^20,24,37–39^.

Detailed demographic and clinical characteristics of all groups can be found in Table 1. There were significant differences in age (*p* = 0.003) and sex (*p* = 0.026) between the five groups. Therefore, both variables were modelled as covariates in the residualisation of the diffusion data and controlled for in all subsequent statistical analyses.

**Table 1:** Group demographics.

	HC (*n* = 59)	ARMS (*n* = 35)	SPT (*n* = 27)	FEP (*n* = 50)	SZ (*n* = 41)	Test	Value	*df*	*p*
Age									
Mean (SD)	29.5 (6.97)	27.4 (9.77)	29.1 (10.4)	26.9 (7.09)	33.7 (9.46)				
Median [min, max]	28.0 [18.0, 54.0]	24.0 [18.0, 64.0]	24.0 [19.0, 48.0]	26.0 [18.0, 47.0]	32.0 [19.0, 52.0]	*F* test	4.23	4	0.003
Sex									
f	28 (47.5%)	10 (28.6%)	10 (37.0%)	10 (20.0%)	10 (24.4%)				
m	31 (52.5%)	25 (71.4%)	17 (63.0%)	40 (80.0%)	31 (75.6%)	${\chi }^{2}$	11.37	4	0.026
Scanner									
BS	24 (40.7%)	35 (100%)	0 (0%)	36 (72.0%)	0 (0%)				
BE	10 (16.9%)	0 (0%)	0 (0%)	0 (0%)	21 (51.2%)	${\chi }^{2}$	157.27	8	<0.001
ZH	25 (42.4%)	0 (0%)	27 (100%)	14 (28.0%)	20 (48.8%)				
IQ									
Mean (SD)	110 (13.0)	113 (15.0)	103 (11.1)	105 (17.5)	101 (11.9)				
Median [min, max]	108 [85.0, 134]	111 [85.0, 139]	97.0 [92.0, 136]	104 [79.0, 143]	97.0 [85.0, 130]	*F* test	2.84	4	0.040
Missing	18 (30.5%)	12 (34.3%)	0 (0%)	13 (26.0%)	24 (58.5%)				
GAF									
Mean (SD)	88.9 (7.34)	70.6 (12.1)	70.9 (11.1)	61.6 (15.6)	54.6 (11.0)				
Median [min, max]	86.0 [61.0, 96.0]	75.5 [46.0, 86.0]	71.0 [50.0, 86.0]	60.0 [16.0, 90.0]	53.0 [35.0, 80.0]	*F* test	26.06	4	<0.001
Missing	33 (55.9%)	3 (8.6%)	0 (0%)	5 (10.0%)	25 (61.0%)				
SANS									
Mean (SD)	0 (0)	14.4 (12.4)	13.2 (6.32)	19.3 (14.7)	21.7 (12.2)				
Median [min, max]	0 [0, 0]	11.0 [0, 41.0]	12.6 [6.33, 25.2]	15.0 [0, 53.0]	18.9 [6.33, 52.5]	*F* test	28.36	4	<0.001
Missing	34 (57.6%)	3 (8.6%)	0 (0%)	11 (22.0%)	15 (36.6%)				
BPRS									
Mean (SD)	24.5 (1.10)	35.5 (6.79)	NA (NA)	46.3 (15.9)	NA (NA)				
Median [min, max]	24.0 [24.0, 28.0]	35.0 [25.0, 57.0]	NA [NA, NA]	47.0 [15.0, 94.0]	NA [NA, NA]	*F* test	29.46	2	<0.001
Missing	35 (59.3%)	3 (8.6%)	27 (100%)	20 (40.0%)	41 (100%)				
Antipsychotics use									
No	59 (100%)	32 (91.4%)	NA	19 (38.0%)	0 (0%)				
Yes	0 (0%)	1 (2.9%)	NA	29 (58.0%)	38 (92.68%)	${\chi }^{2}$	305.27	8	<0.001
Missing	0 (0%)	2 (5.7%)	27 (100%)	2 (4.0%)	3 (7.32%)				
Antidepressant use									
No	59 (100%)	23 (65.7%)	NA	26 (52.0%)	12 (29.3%)				
Yes	0 (0%)	9 (25.7%)	NA	12 (24.0%)	5 (12.2%)	${\chi }^{2}$	138.28	8	<0.001
Missing	0 (0%)	3 (8.6%)	27 (100%)	12 (24.0%)	24 (58.5%)				

*Note*. Data are presented as means and SDs, Median and minimum and maximum. *HC* healthy control; *ARMS* at risk mental state; *FEP* first episode psychosis; *SPT* schizotypal personality traits; *SZ* patients with schizophrenia; *IQ* Multiple Word Test Intelligence Quotient; *GAF* Global Assessment of Functioning; *SANS* Scale for Assessment of Negative Symptoms; *BPRS* Brief Psychiatric Rating Scale. *p*-values FDR corrected.

### Image acquisition and preprocessing of diffusion derived data

Details of diffusion imaging sequences can be found in the supplement (Supplementary Table 1). Data quality assessment and preprocessing were performed as previously done and described in detail^20^ (see supplement). The quality inspection resulted in the exclusion of 10 subjects (2 HC, 3 ARMS, 5 FEP). From the resulting preprocessed datasets, the fractional anisotropy (FA) and mean diffusivity (MD) tensor related measures were derived using the FSL software package. Furthermore, fibre density (FD), a non-tensor-derived diffusion marker, measure was calculated as previously described in detail^20^. Preprocessing has been performed as previously described^20,26,39^ (for details see supplement).

### Harmonisation

Site-related differences in the FA, MD, and FD measurements were controlled with the ComBat harmonisation technique which has shown to remove unwanted variation in DTI data induced by site^51^. Diffusion imaging data were harmonised prior to heterogeneity analyses controlling for age, sex, and group. ComBat was first developed as a batch adjustment method for genomics data^52^ and later adapted by Fortin et al.^51^ in the context of DTI images. The generalised ComBat model has demonstrated to successfully reduce inter-site effects for voxels in the FA, MD, and FD maps and fully remove site effects for all regions of interest (ROIs). They further showed that the use of Empirical Bayes implemented in ComBat improved the estimation and removal of site-effects, making this approach especially robust to harmonise data, whilst preserving the between-subject variability in small sample size studies^51^. The ComBat harmonisation was implemented in R using the software provided on GitHub (https://github.com/Jfortin1/ComBatHarmonization).

To further account for potentially persisting confounding effects of scanner, age, and sex on the diffusion parameters, residualisation as described in previous studies^53,54^ was performed (for details see supplement).

### Calculation of global heterogeneity

The PBSI is a validated method which allows for quantification of individual neuroimaging profiles and their similarity to those of other members of their group^17,55,56^. A three-step procedure was used to derive PBSI scores by collapsing all WM tracts and creating FA, MD, and FD profiles for each subject. Subsequently interindividual Spearman’s correlation coefficients were calculated between each of the subjects and the respective profile of the other subjects. Lastly the *n*-1 interindividual correlation coefficients (*n* referring to the number of subjects in each sample) were averaged separately resulting in one PBSI score with a maximum of 1 per participant^17,55,56^. The higher the PBSI score the greater the similarity between the diffusion parameter (FA, MD, or FD) profiles of the subject with those of the same sample. The MATLAB function used to compute the PBSI scores is available at: https://www.mathworks.com/matlabcentral/fileexchange/69158-similarityscore^57^:

$${{\rm{PBSI}}}_{i}=\frac{1}{N-1}\sum _{j\ne i}{\rm{cor}}({y}_{i},{y}_{j})$$

In this study the PBSI scores were computed separately for FA, MD, and FD maps to generate a person-specific neuroimaging profile in correspondence to profiles of members of the same group (PBSI, within-group reference) and individually in reference to the neuroimaging profile of a normative reference group regardless of continuum stage (nPBSI, normative reference). The respective scores provide an estimate of the similarity of the individual neuroimaging profiles indicating the degree of heterogeneity within the groups (PBSI) and in reference to the norm (nPBSI).

### Within-group reference

The PBSI_FA_, PBSI_MD_ and PBSI_FD_ were calculated separately for the five groups (HC, ARMS, FEP, SPT and SZ) representing the degree of within-group profile similarity. For each individual group the three-step procedure of calculating PBSI were performed. The higher the PBSI score the greater the similarity in the neuroanatomical profile of the individual respective to its group. The within-group reference represents the level of heterogeneity of each individual to other members of the same group.

### Normative reference

To quantify the similarity of the individuals’ neuroanatomical profile respective to a normative estimate the nPBSI_FA_, nPBSI_MD_, and nPBSI_FD_ scores were calculated for each individual subject from the four clinical groups (ARMS, FEP, SPT, SZ) in correlation with the corresponding profiles of the control group (HC). The nPBSI scores resulting from three-step procedure indicate the degree of deviation from the normative range^16^.

### Statistical analysis of global heterogeneity

Differences of PBSI_FA,_ PBSI_MD,_ and PBSI_FD_ scores between the five groups were assessed using analysis of variance (ANOVA) and Welch’s test proven to be robust for unequal sample sizes^58,59^. Statistically significant results were followed up with post-hoc pairwise comparisons to examine differences between specific groups.

The nPBSI scores for FA, MD, and FD of each group were converted into z-scores (z-nPBSI) representing the deviation from the mean of the healthy control group (PBSI-HC). To identify the deviators from the norm the threshold of ±1.5 SD was applied^16,18^. To determine whether the number of deviators differed between the groups, for each diffusion parameter Fishers’s exact test was performed, and the odds ratio (OR) determined for effect size. T-tests were performed to compare clinical measures between deviators and non-deviators and Mann–Whitney *U* used to examine the effect of group specific deviators. Subsequently Pearson’s correlation coefficients were calculated to evaluate correlations between deviators and non-deviators with crystallised intelligence (IQ), global functioning (GAF) and clinical scores (SANS, BPRS). This correlation was computed separately for every parameter (FA, MD, and FD) and overall measures (IQ, GAF, SANS & BPRS). To account for multiple comparisons, all *p*-values were adjusted using false discovery rate; when uncorrected *P* values are shown these are denoted as p_unc_. All statistical analyses were conducted using R Studio (version 2023.03.0) within the R programming environment (version 4.1.2 and 4.3.1).

### Calculation of local heterogeneity

**Fig. 1:**
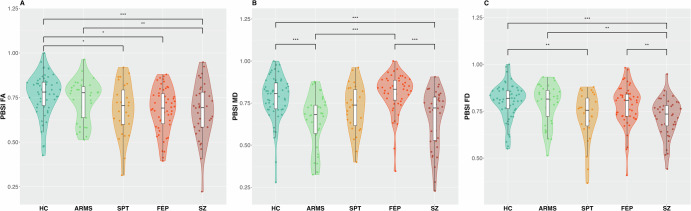
Within group PBSI scores per parameter. Person-based-similarity-index (PBSI) for fractional anisotropy (**A**), mean diffusivity (**B**), and fibre density (**C**) in healthy controls (HC), at-risk-mental-state (ARMS), schizotypy (SPT), first-episode psychosis (FEP), and schizophrenia (SZ). ANCOVA analysis controlling for age and sex followed up by estimated marginal means (EMMs) for pairwise-comparisons, * <0.05, ** <0.01, *** <0.001 = sign. after FDR correction.

**Fig. 2:**
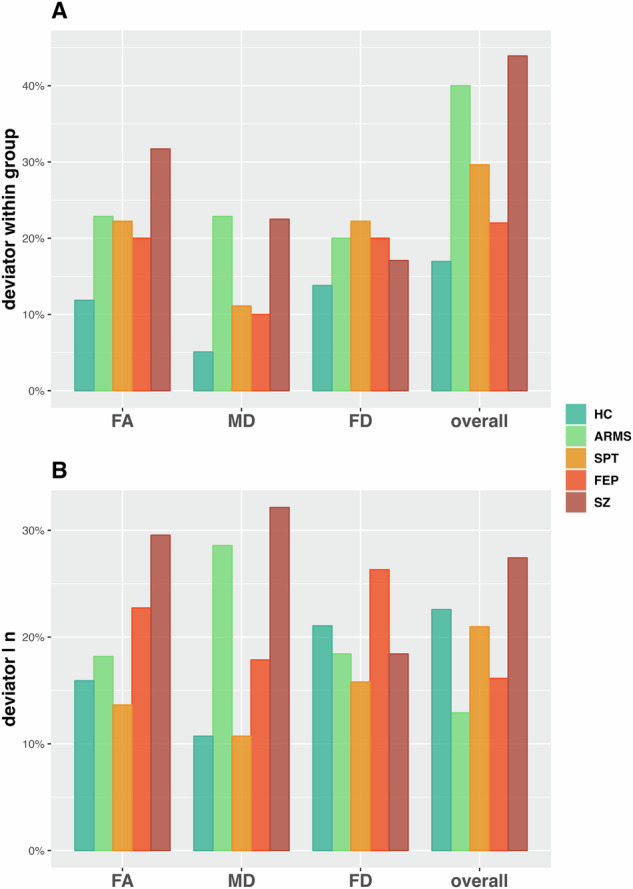
Within group and parameter deviator ratios. Bar chart representing the percentage of individuals within the individual groups (HC, ARMS, SPT, FEP & SZ) who demonstrate marked deviation of the person-based similarity index cortical thickness z-scores from the ‘normative’ neuroanatomical profile (±1.5 SD), per parameter and overall (**A**). Bar chart representing the percentage of deviators accounted for by individual groups relative to group size, per parameter and overall (**B**).

For each parameter and WM tract the coefficient of variation ratio (CVR) was computed across groups to estimate differences in local heterogeneity. We used the escalc() function in R^60^ to apply the log-CVR function as a mean-scaled variability measure of diffusion parameters between two groups. It is calculated using the following formula:

$${\mathrm{ln}}{CVR}={\mathrm{ln}}\left(\frac{{\hat{\sigma }}_{p}/{\bar{{x}}}_{p}}{{\hat{\sigma }}_{c}/{\bar{{x}}}_{c}}\right)={\mathrm{ln}}\left(\frac{\frac{{S}_{p}}{{\bar{{x}}}_{p}}}{\frac{{S}_{c}}{{\bar{{x}}}_{c}}}\right)+\frac{1}{2\left({n}_{p}-1\right)}-\frac{1}{2\left({n}_{c}-1\right)}$$

where x_p_ and x_c_ indicate the reported means for the clinical (ARMS, FEP, SPT, and SZ) and control (HC) group, respectively; the ${\hat{\sigma }}_{p}$ and ${\hat{\sigma }}_{c}$ indicate the unbiased estimates of population SDs; S_p_ and S_c_ represent the reported sample SDs; n_p_ and n_c_ are the sample sizes. Next, the difference in variability was computed in the same manner between the groups FEP vs. SZ, and ARMS vs FEP. The log CVR was back-transformed into the original scale in order to simplify the interpretation of the results. A CVR of 1 indicates equal variability of diffusion parameters in the WM tracts between the groups, whereas a CVR < 1 indicates a smaller variability in the control group, and CVR > 1 indicates a greater variability in the more symptomatic group, when compared to a less symptomatic group. The results are shown in forestplots (see Figs. 1 and 2 and Supplementary Figs. 2–16), showing the corrected as well as the uncorrected *p*-values. For *p*-value adjustment, we used the False Discovery Rate (FDR) method.

## Results

### Group differences in global heterogeneity

Across groups significant PBSI differences were found for all three diffusion measures FA (*F* (1,4) = 5.33, *p* < 0.001), MD (*F* (1,4) = 6.61, *p* < 0.001), and FD (*F* (1,4) = 4.91, *p* = 0.001). Specific group differences in PBSI_FA_ were found to be most pronounced between SZ-HC (*t* = −4.37, *p* = <0.001), SZ-ARMS (*t* = −3.016, *p* = 0.012), FEP-HC (*t* = −2.93, *p* = 0.012), and SPT-HC (*t* = −2.39, *p* = 0.045) (Fig. 1A). For PBSI_MD_, significant differences were found between SZ-HC (*t* = −3.85, *p* < 0.001), SZ-FEP (*t* = −3.96, *p* < 0.001), FEP-ARMS (*t* = 3.92, *p* < 0.001), ARMS-HC (*t* = −3.80, *p* < 0.001) (Fig. 1B). For PBSI_FD_ differences were found between SZ-HC (*t* = −3.96, *p* = 0.001), SZ-ARMS (*t* = −3.35, *p* = 0.005), SZ-FEP (*t* = −3.06, *p* = 0.006), SPT-HC (*t* = −3.08, *p* = 0.006), SPT-ARMS (*t* = −2.47, *p* = 0.029), and trends were found between FEP-SPT (*t* = 2.18, *p* = 0.051) (Fig. 1C). For details on group comparisons see Supplementary Table 2.

### Normative referencing of global heterogeneity

Over all three parameters the number of deviators differed significantly across the groups (${\chi }^{2}$ = 11.86, df = 4, *p* = 0.018). Subsequent post-hoc comparisons revealed significant differences between the groups SZ-HC (OR = 3.78, *p* = 0.006), SZ-FEP (OR = 2.74, *p* = 0.041), and ARMS-HC (OR = 3.22, *p* = 0.016), yet notwithstanding correction for multiple comparisons.

Within group, SZ showed the greatest percentage of deviators (31.71%) followed by ARMS (22.86%), SPT (22.22%), FEP (20.00%), and HC (11.86%) for FA. For MD, 22.50% deviators were found in SZ, 22.86% in ARMS, 11.11% in SPT, 10.00% in FEP, and 5.09% in HC. And for FD 22.22% in SPT, 20.00% in FEP and ARMS, 17.07% in SZ, and 13.79% in HC (Fig. 2A).

Across all groups SZ accounted for the most deviators (29.55%) followed by FEP (22.73%), ARMS (18.18%), HC (15.91%), and SPT (13.64%) for FA. For MD most deviators were accounted for by SZ (32.14%) followed by ARMS (28.57%), FEP (17.86%), SPT (10.71%), and HC (10.71%). And for FD 26.32% in FEP, followed by 21.05% in HC, 18.42% in SZ and ARMS, and 15.79% in SPT (Fig. 2B).

Further, for nPBSI_MD_ a significant difference in the number of deviators (${\chi }^{2}$ = 9.76, df = 4, *p* = 0.041) was discovered across all groups. Subsequent post-hoc comparisons revealed differences between SZ-HC (OR = 5.32, *p* = 0.013), and ARMS-HC (OR = 5.42, *p* = 0.017), again notwithstanding correction for multiple comparisons. For for nPBSI_FA_ (${\chi }^{2}$ = 5.97, df = 4, *p* = 0.188), and nPBSI_FD_ (${\chi }^{2}$ = 1.28, df = 4, *p* = 0.847) no significant differences in deviators from the norm were detected.

### Deviation from the normative reference and clinical implications

**Fig. 3:**
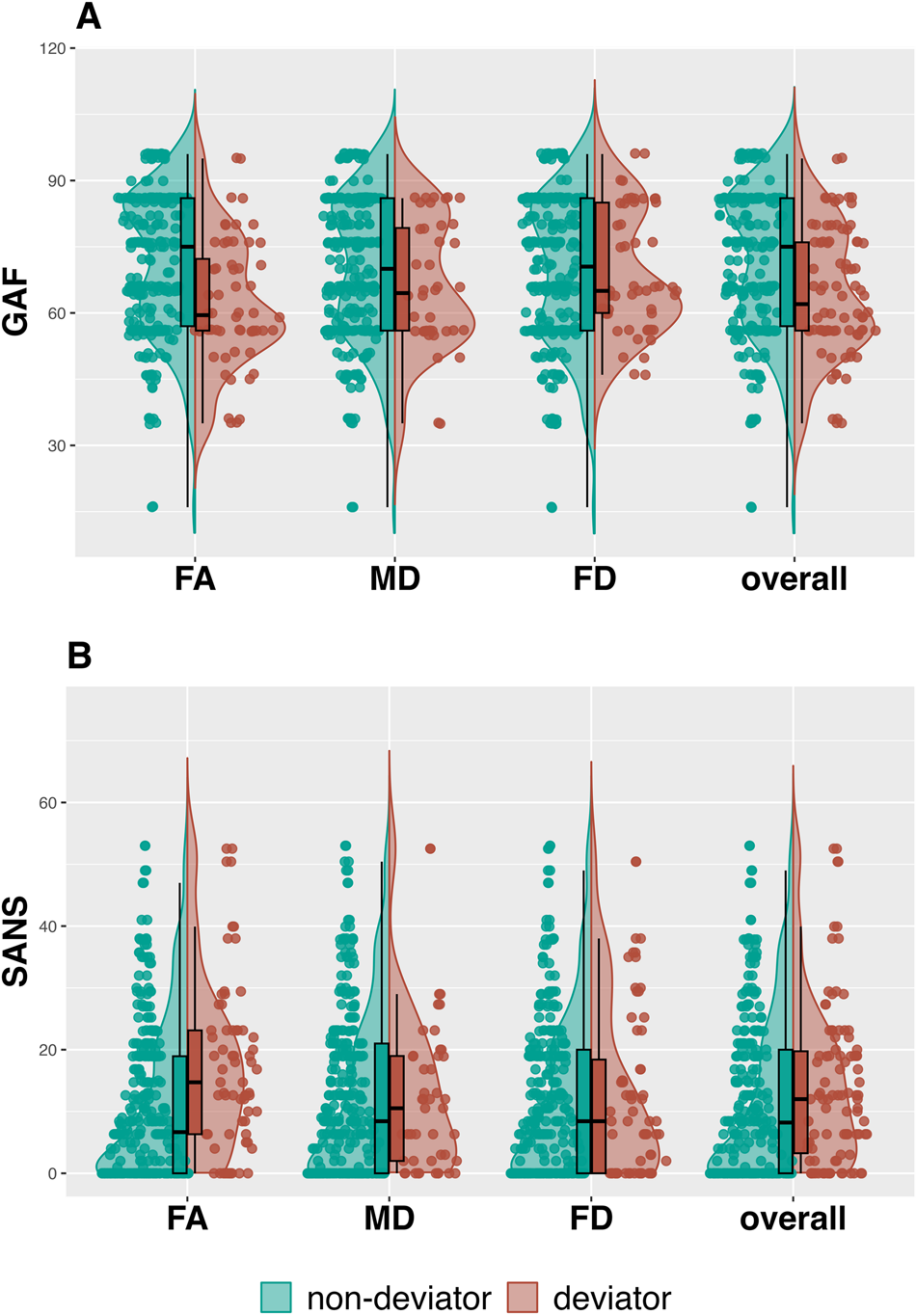
Differences in GAF and SANS between deviators. Deviators vs. non-deviators for nPBSI_FA_, nPBSI_MD_, and nPBSI_FD,_ and overall parameters in general ability of functioning (GAF) (**A**), and scale for the assessment of negative symptoms (SANS) (**B**) scores.

The analysis of the relationship between being a deviator (vs non-deviator), crystallised intelligence (IQ), generalised functioning (GAF) and psychopathology (SANS, BPRS) underpins the connection between FA deviators in GAF (*t* = 2.64, *p*_*unc*_ = 0.009) and SANS (*t* = −2.43, *p*_*unc*_ = 0.016), indicating towards lower GAF scores, and higher SANS scores in FA deviators (Fig. 3B). However, these results are notwithstanding correction for multiple comparisons. No significant correlation for deviators in specific parameters and clinical features could be found for the other measures.

### Group differences in local heterogeneity

Only the FDR-corrected statistically significant between-group differences in local heterogeneity will be reported here.

### Fractional anisotropy (FA)

**Fig. 4:**
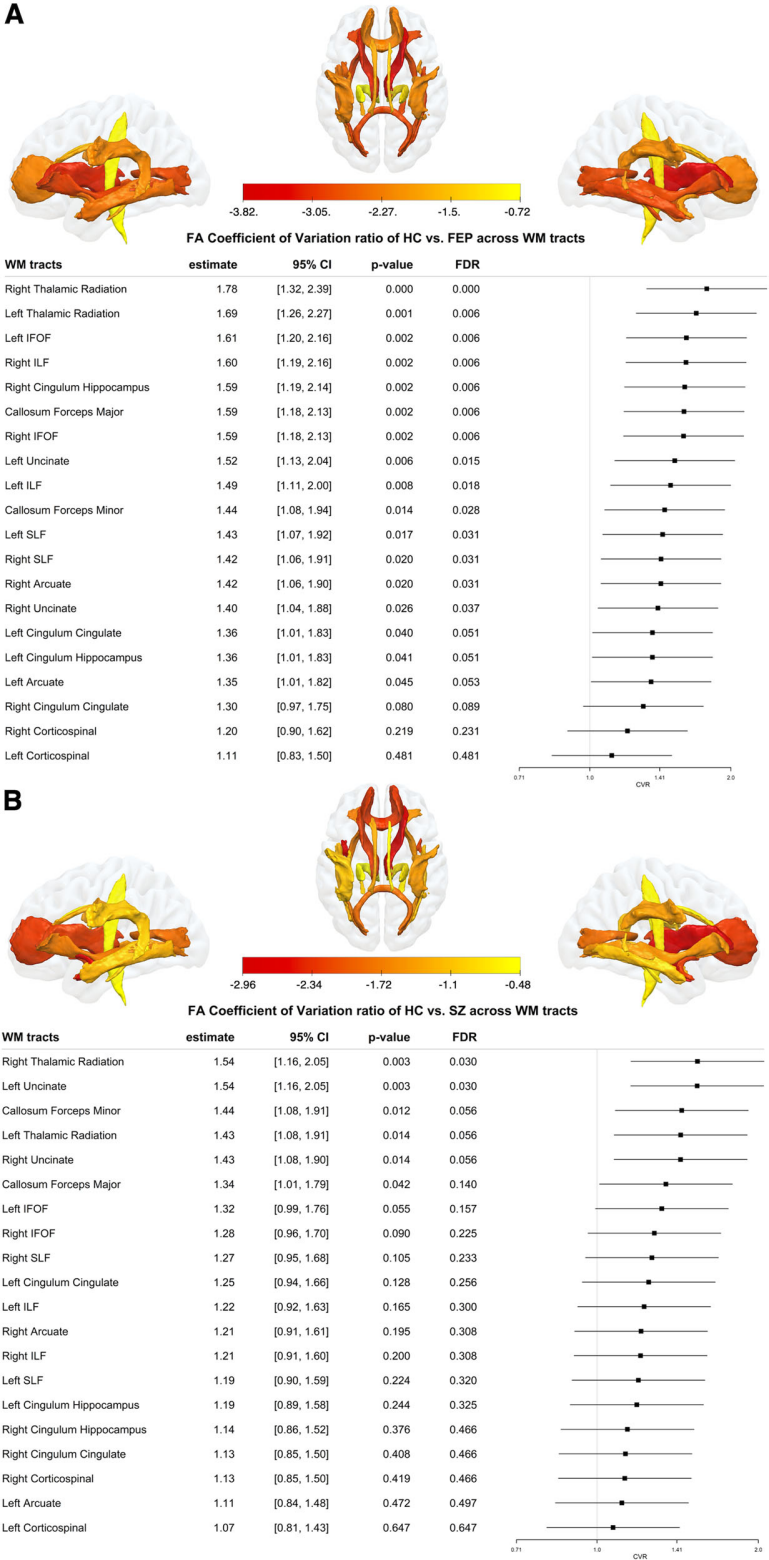
Variability in local WM heterogeneity of FA. Forest plots of the coefficient of variation ratio (CVR) of fractional anisotropy (FA) between (**A**) healthy controls (HC) and first episode psychosis (FEP), and (**B**) between HC and diagnosed schizophrenia (SZ). Colour gradient indicating CVR effect sizes transformed into z-scores for the individual tracts.

As illustrated in Fig. 4A, FEP showed a greater heterogeneity in all WM tracts, except both corticospinal, both cingulum cingulate, left cingulum hippocampus and left arcuate WM tracts, when compared to HC. SZ showed a greater heterogeneity in the right thalamic radiation and the left uncinate WM tract (Fig. 4B), when compared to HC. Also, FEP showed greater heterogeneity than ARMS in all WM tracts, besides the left corticospinal tract. The comparisons of HC vs. ARMS, HC. vs. SPT, and FEP vs. SZ did not show any differences in CVR in any WM tracts when compared to HC (Supplementary Figs. 2–5).

### Mean diffusivity (MD)

FEP showed greater heterogeneity in the left cingulum hippocampus and the right thalamic radiation WM tract, when compared to HC. Also, SZ showed greater heterogeneity in the right thalamic radiation WM tract than the HC group. There were no effects in ARMS vs. HC, MD for FEP vs. ARMS, SPT vs. HC and FEP vs. SZ in any WM tracts (Supplementary Figs. 6–11).

### Fibre density (FD)

None of the local heterogeneity group comparisons showed any statistically significant differences in any of the WM tracts after FDR-correction (Supplementary Figs. 12–16).

## Discussion

This study revealed four major findings: firstly, global heterogeneity significantly differed across groups and diffusion parameters. The greatest difference in global heterogeneity was apparent between SZ and HC across all three diffusion parameters. ARMS additionally showed greater heterogeneity in MD relative to HC, SPT, and FEP, yet not SZ, whereas in FA and FD, ARMS differed from SZ. SPT revealed to differ markedly from SZ (FA), ARMS, and SZ (MD), as well as ARMS and HC (FD), yet not FEP. Secondly, the number of deviators differed significantly between groups for MD but not for FA and FD. In particular, MD deviators were highest in SZ (9), followed by FEP (5), ARMS (8), HC (3), and SPT (3) relative to sample size. Thirdly, deviators in FA indicated towards lower GAF, and higher SANS scores, respectively. Finally, greater local heterogeneity (i.e. CVR) in FA was found in almost all WM tracts in FEP relative to HC and ARMS, while SZ patients showed greater local heterogeneity specifically in the right thalamic radiation and the left uncinate when compared to HCs. For MD and in relation to HC, FEP, and SZ patients revealed greater heterogeneity in the right thalamic radiation, and FEP additionally in the left cingulum hippocampus. Differences in WM heterogeneity was observed in global and local measures for all explored diffusion parameters across the psychosis continuum.

### Global within-group heterogeneity across groups

The analysis of PBSI scores revealed significant global within-group heterogeneity, particularly in the SZ group, and highlighted marked differences between SZ-HC, SZ-ARMS, FEP-HC, and SPT-HC. PBSI_FD_ showed similar patterns but also indicated greater heterogeneity between SPT and ARMS, as well as HC, while FEP did not differ significantly from HC and ARMS. In contrast, PBSI_MD_ revealed higher heterogeneity in ARMS and SZ compared to HC, with additional differences observed between SPT-ARMS, SPT-SZ, and FEP-ARMS, FEP-SZ (Fig. 1).

### ARMS and clinical implications

The lack of PBSI_FA_ differences in the ARMS group aligns with previous studies reporting no significant mean FA differences between HC and ARMS^23,26^. However, evidence suggests that at-risk individuals who convert to full-blown psychosis often show reduced FA, potentially contributing to variability^23,26,61^. In this study, the small sample size and number of converters (*n* = 6, mean follow-up of 3.8 years) limited the ability to conduct a meaningful subanalysis.

Interestingly, while no reduction in PBSI_FA_ was observed in ARMS, a reduction in PBSI_MD_ was noted, corresponding to previously reported increases in MD^23^. This greater global heterogeneity in MD is consistent with recent studies showing that ARMS also exhibit lower PBSI scores for structural measures such as cortical thickness (CT), surface area (SA), and subcortical volume (SV)^16,18^. These findings suggest that the clinical heterogeneity in ARMS^7^ may be better reflected in increased global heterogeneity in MD or morphological metrics rather than in FA.

### FEP and HC comparisons

The comparable PBSI_MD_ and PBSI_FD_ scores between HC and FEP are notable, especially given the significant reductions in mean FA^62^ observed in the FEP group. This finding is consistent with the lack of global heterogeneity in morphological metrics reported in patients with bipolar disorder^56^. It is possible that the global heterogeneity of diffusion markers in FEP patients^56^ may fall within the range of normal biological diversity, similar to the high prevalence of psychotic symptoms in the general population. However, this interpretation should be approached with caution, given the small sample size of our FEP group and the pronounced increases in local heterogeneity measures observed in FEP patients.

### SZ, SPT, and clinical trajectories

The increased global heterogeneity across all diffusion parameters in SZ aligns with previous evidence of increased global heterogeneity in morphological metrics (SA, CT, SV)^63,64^ and expands the understanding of mean diffusion differences in SZ^19,20,65^. This increased heterogeneity may be attributed to the high clinical heterogeneity in SZ^13^. Similarly, SPT individuals showed greater global heterogeneity in FA and FD parameters compared to HC, mirroring the pattern observed in SZ^66^. Both SZ and SPT groups are characterised by highly variable longitudinal trajectories^13,15,67^, suggesting that dissecting variability in established diffusion measures could help identify subgroups of SPT and SZ patients with distinct clinical trajectories, ranging from more severe (e.g., reactive compensation or resilience) to milder (e.g., relapses) courses^68^.

### Normative referencing for global heterogeneity

Despite significant differences in the number of normative heterogeneity deviators across the five groups, no statistically significant correlations were found between these deviations and verbal intelligence (IQ), global functioning (GAF), or clinical symptoms (SANS, BPRS) across all groups. As in previous studies^69^, symptom severity was not associated with deviations from the normative reference. However, consistent with prior research^26,35^ and our hypothesis, differences in the number of deviators, were observed for nPBSI_MD_, but not for nPBSI_FA_ or nPBSI_FD_.

The investigation into the relationship between deviators from the ARMS and FEP groups with IQ and GAF scores, which have been suggested in earlier research on GMV heterogeneity^18^, did not reflect similar findings in white matter (WM) deviators. Nonetheless, the negative correlations observed between FA deviators and GAF scores are in line with findings by Antoniades et al.^18^, which linked higher psychopathology to deviators in individuals at clinical high risk (CHR) and those experiencing first-episode psychosis (FEP).

The observation of higher SANS scores in FA deviators aligns with previous research demonstrating a significant relationship between negative symptoms in CHR and multimodal changes in brain morphometry, including increased regional FA in CHR individuals who transitioned to psychosis^70^. A longitudinal follow-up study with the same individuals could help determine which parameters and respective PBSI scores and nPBSI deviators have the potential to serve as predictive indicators of disease risk.

Previous large-scale investigations into brain morphology (SA, CT, SV) did not find a link between deviations in CHR from a normative reference and psychopathology, nor did they associate these deviations with the transition to psychosis^16^. Similarly, another large-scale case-control study examining the same morphometric measures did not find an association between regional deviations and the severity of positive symptoms or IQ, suggesting that other structural metrics, such as DTI, may better distinguish between stages of psychosis^71^. Our findings indicate strong trends suggesting that FA deviators may differentiate individuals with schizophrenia (SZ) from healthy controls (HC), as well as those at ARMS and individuals experiencing FEP. While region-specific deviant DTI measures do not seem helpful for SZ-HC classification^72^, larger studies are needed to determine whether global diffusion heterogeneity can identify subgroups with common psychopathology.

In line with recent studies on GMV heterogeneity, we hypothesised that clinical measures such as IQ scores would positively correlate with PBSI scores^64^. Although white matter tract integrity has been shown to explain 10% of intelligence differences^73^, our sample did not show differences in IQ relative to their deviance status. This may be attributed to the nature of the IQ test (MWTB) used in this study, which lacks time constraints and thus does not account for information processing speed—a factor known to play a vital role in general functioning^73^. Consequently, more comprehensive evaluations of general functioning should be conducted in the future. The significant results indicating lower GAF scores in deviators suggest a link to general functioning, which could be further specified with more differentiated tests.

### Local WM heterogeneity

On a local scale, the FA parameter exhibits the most statistically significant tract-specific differences in heterogeneity among group comparisons. Despite its previously demonstrated specificity for early-stage psychosis^26,34,35^, the FA parameter did not show statistically significant differences in local heterogeneity between HC-ARMS or HC-SPT, which are considered early stages on the psychosis risk continuum. Significant local differences in white matter (WM) heterogeneity were observed only between HC-FEP, HC-SZ, and ARMS-FEP.

The FEP group, which exhibited a greater number of WM tracts with significant heterogeneity compared to HC, contrasts with the SZ group, which showed fewer tracts with such distinctions. This discrepancy could potentially be explained by patterns of volume loss, particularly in the thalamus, where reductions in specific nuclei vary depending on the stage of disease progression^74^. For instance, medial nuclei reductions are suggested to occur earlier, while lateral nuclei impairments emerge at later stages. Consequently, the more widespread heterogeneity observed in FEP could result from the dynamic progression of psychopathology, with consistently changing symptoms, whereas SZ, representing a more stabilised disease state, may display less extensive heterogeneity.

The possibility that these differences are solely due to antipsychotic effects seems unlikely, as recent research has shown higher variability in WM tracts among individuals with FEP before the initiation of antipsychotic treatment^75^. The persistent FA abnormalities in SZ may indicate the presence of different schizophrenia subtypes. The higher prevalence of FA differences in heterogeneity, compared to MD and FD, aligns with previous research that reported the largest effect sizes for lower average FA in 80% of the investigated ROIs in schizophrenia^19^. While MD has been associated with increased values in patients, our findings suggest that the decrease in FA affects a broader range of WM tracts, while the increase in MD, occurring alongside changes in FD, follows a similar pattern but in fewer tracts^19^.

The lack of local heterogeneity differences between HC and ARMS is consistent with findings in GMV, where no regional differences in SA, CT, or SV heterogeneity were observed between HC and CHR^16^. Local variability differences may become evident only when symptom severity reaches the level of FEP, potentially allowing us to discern underlying biological processes that point to distinct biological subtypes^14^. The greater local WM heterogeneity observed in our study is consistent with previous research on grey matter heterogeneity, which suggests that collapsing patients with differing biological signatures into a single diagnostic group^13^ may lead to increased variability. Our results align with recent reports on structural GM heterogeneity, yet further investigation is needed to explore the potential of local heterogeneity as a marker for subtype delineation^15^.

### Limitations

Although our study covered several stages on the psychosis risk spectrum more large-scale longitudinal studies are required to ascertain how WM heterogeneity can differentiate between underlying subtypes.

The small sample size relative to the number of centres may have introduced scanner effects that COMBAT harmonisation could not fully address, and had to be further residualised for differences in HC across scanners, which should be considered when interpreting the results. Although images with significant motion artifacts were excluded, the greater variability observed in the patient and psychosis risk groups might still reflect residual movement-related noise. Additionally, since the groups were not evenly distributed across scanners, the results should be interpreted with caution. As this was a post-hoc study, we were able to include some common measures of crystallised intelligence, global functioning, and psychopathology, yet due to sample size constraints and missing data we could not draw conclusions regarding medication. More homogeneous and comprehensive cognitive test batteries, comprehensive information of medication, and clinical evaluation across all groups are required to yield conclusive results on the effects of WM heterogeneity on clinical symptoms. More data driven studies would allow for an unconfined subgroup analysis and normative modelling which hold the potential for predictive risk assessments, and thus could inspire novel paths to disease prevention.

## Conclusion

This study highlights differences in global and local white matter heterogeneity across the psychosis continuum, with the most pronounced differences observed between schizophrenia (SZ) and healthy controls (HC) for all diffusion parameters. The number of deviators in mean diffusivity (MD) differed significantly across groups, with SZ exhibiting the highest number. Local heterogeneity in white matter tracts was particularly notable in FEP and SZ, indicating potential variations in disease trajectories. These findings support the heuristic framework of staging models which emphasise that the developmental trajectory of psychosis is neither fixed nor linear^10^ and underscore the importance of considering global and local variability in understanding psychosis-related brain changes. While these findings offer valuable insights into the neurobiological variability of psychosis, they are primarily hypothesis-generating for future research. Larger samples and more comprehensive cognitive assessments are needed to validate these observations and explore potential clinical subtypes.

## Supplementary information

Revised supplement - clean

## Data Availability

The data that support the findings of this study and code to reproduce the results shown in the paper are available from the corresponding author upon reasonable request. We used R version 4.1.2 and 4.3.1.
