# Supplementary material for: Parsing heterogeneity in global and local white matter integrity at different stages across the psychosis continuum
Source: Schizophrenia (Heidelb). 2024 Nov 13;10(1):106. doi: 10.1038/s41537-024-00516-7 (PMC11561281; doi:10.1038/s41537-024-00516-7)
Supplement: Supplementary file 1 — Revised supplement - clean [file 41537_2024_516_MOESM1_ESM.docx]

**Supplementary Material**

**Supplement**

**Image acquisition and preprocessing of diffusion derived data**

Diffusion data were first corrected for effects of motion and image distortions due to eddy currents ^1^ using FMRIB Software Library (FSL, version 5.0.7, [www.fmrib.ox.ac.uk/fsl](http://www.fmrib.ox.ac.uk/fsl)). The brain extraction tool (BET) ^2^ was then applied to remove non-brain tissue from the diffusion data and to estimate the inner and outer skull surfaces. Additionally, residual susceptibility-induced distortions caused by EPI sequences were corrected by the bdp algorithm in BrainSuite ^3^ incorporating T1-weighted MPRAGE images as reference. Subsequently, the following quality criteria have been used: First diffusion tensor residuals were calculated for every acquired diffusion direction and the nine slices in the whole diffusion dataset with the highest residuals were identified for visual inspection. Second, the MRtrix3 software package (<http://www.mrtrix.org>) was used to estimate the voxel-wise noise using the residuals from a truncated spherical harmonics fit. Plots were generated depicting the twelve slices with the highest noise level, four in sagittal, four in axial, and four in coronal direction, respectively. Third, mean signal intensity plots for every diffusion direction and the non-diffusion-weighted image were derived and plotted slice by slice in sagittal, axial, and coronal directions. Artifacts such as signal dropouts due to head motion can easily be spotted on these plots. Two trained MR physicists separately inspected the data for artefacts and rated the signal courses and fitting residuals of every subject on a Likert-type scale. Based on these criteria 10 subjects had to be excluded (2 HC, 3 ARMS, 5 FEP). Finally, the tensor fitting, and computation of four tensor metrics were performed. FA was computed as the variance among the three orthogonal eigenvectors of diffusion tensors ^4,5^ and is considered a proxy of WM integrity and most probably reflects its geometrical properties that may be influenced by the geometrical fiber configuration, density, and axon diameter, as well as myelination ^6^. MD is an overall and rotationally invariant diffusion marker ^7^ and FD a parameter related to restricted intra-axonal compartment ^8^.

**Cross scanner harmonization**

After conducting COMBAT to account for site-specific difference to account for potentially remaining confounding effects we residualised the PBSI and nPBSI data. First, the dataset was subset to include only the healthy control (HC) group. A series of linear regression models were then fitted for each parameter FA, MD, and FD using scanner, age, and sex as predictors. The resulting models, derived from the HC group, were subsequently used to predict values for the entire dataset. The residuals were calculated by subtracting these predicted values from the observed values for each participant. These residuals, representing the variance in the diffusion parameters not explained by scanner, age, or sex, were used for further analysis^9-11^.

**Supplementary Table 1.**

*Imaging parameters per site.*

| **Site** | Model | Field strength | num. dir. | b-values/  reference | TR (ms) | TE (ms) | slice num | slice thick.  (mm) | voxel  (mm^3^) |
| --- | --- | --- | --- | --- | --- | --- | --- | --- | --- |
| **Basel** | SIEMENS  Magnetom Verio, Siemens Healthcare, Erlangen, Germany | 3T | 30 | 900s/mm^2^  single:  b=0s/mm^2^ | 9200 | 95 | 54 | 2.5 | 2.5 x 2.5 x 2.5 |
| **Bern** | SIEMENS  Magnetom Verio, Siemens Healthcare, Erlangen, Germany | 3T | 42 | 1300s/mm^2^  min.  b=0s/mm^2^ | 9100 | 88 | 55 | 2.2 | 2 x 2 x 2.2 |
| **Zurich** | Philipps  Achieva, Philips Healthcare, Best, the Netherlands | 3T | 32 | 1000s/mm^2^  four:  b=0s/mm^2^ | 6640 | 53.6 | 50 | 2.5 | 2.5 x 2.5 x 2.5 |

**Supplementary Table 2.**

*Group differences in global heterogeneity*

| FA: | ARMS | FEP | SPT | SZ |
| --- | --- | --- | --- | --- |
| HC | 0.406 | 0.012* | 0.045* | <0.001* |
| ARMS | **-** | 0.188 | 0.226 | 0.012* |
| FEP | **-** | **-** | 0.968 | 0.211 |
| SPT | **-** | **-** | **-** | 0.226 |
| MD: | **ARMS** | **FEP** | **SPT** | **SZ** |
| HC | <0.001* | 0.870 | 0.238 | <0.001* |
| ARMS | - | <0.001* | 0.075 | 0.921 |
| FEP | - | - | 0.226 | <0.001* |
| SPT | - | - | - | 0.075 |
| FD: | **ARMS** | **FEP** | **SPT** | **SZ** |
| HC | 0.574 | 0.417 | 0.006* | 0.001* |
| ARMS | **-** | 0.769 | 0.067 | 0.005* |
| FEP | **-** | **-** | 0.074 | 0.005* |
| SPT | **-** | **-** | **-** | 0.533 |

**Supplementary Table 3**

*Contingency tables for nPBSI*

| **FA** | **Non-deviator** | **Deviator** | **n** |
| --- | --- | --- | --- |
| **HC** | 52 | 7 | 59 |
| **ARMS** | 27 | 8 | 35 |
| **SPT** | 21 | 6 | 27 |
| **FEP** | 40 | 10 | 50 |
| **SZ** | 28 | 13 | 41 |

| **MD** | **Non-deviator** | **Deviator** | **n** |
| --- | --- | --- | --- |
| **HC** | 56 | 3 | 59 |
| **ARMS** | 27 | 8 | 35 |
| **SPT** | 24 | 3 | 27 |
| **FEP** | 45 | 5 | 50 |
| **SZ** | 31 | 9 | 40 |

| **FD** | **Non-deviator** | **Deviator** | **n** |
| --- | --- | --- | --- |
| **HC** | 50 | 8 | 59 |
| **ARMS** | 28 | 7 | 35 |
| **SPT** | 21 | 6 | 27 |
| **FEP** | 40 | 10 | 50 |
| **SZ** | 34 | 7 | 41 |

| **overall** | **Non-deviator** | **Deviator** | **n** |
| --- | --- | --- | --- |
| **HC** | 49 | 10 | 59 |
| **ARMS** | 21 | 14 | 35 |
| **SPT** | 19 | 8 | 27 |
| **FEP** | 39 | 11 | 50 |
| **SZ** | 23 | 18 | 41 |

**Supplementary Figure 1.Supplementary Figure 2.**

**

**Supplementary Figure 3.**

**Supplementary Figure 4.**

**Supplementary Figure 5.**

**Supplementary Figure 6.**

**Supplementary Figure 7.**

**Supplementary Figure 8.**

**Supplementary Figure 9.**

**Supplementary Figure 10.**

**

**Supplementary Figure 11.**

**Supplementary Figure 12.**

**Supplementary Figure 13.**

**Supplementary Figure 14.**

**Supplementary Figure 15.**

**Supplementary Figure 16.**

1. Andersson JLR, Sotiropoulos SN. An integrated approach to correction for off-resonance effects and subject movement in diffusion MR imaging. *Neuroimage*. Jan 15 2016;125:1063-1078. doi:10.1016/j.neuroimage.2015.10.019

2. Smith SM. Fast Robust Automated Brain Extraction. Human Brain Mapping. *Hum Brain Mapping*. 2002;17:143-155.

3. Bhushan C, Haldar JP, Choi S, Joshi AA, Shattuck DW, Leahy RM. Co-registration and distortion correction of diffusion and anatomical images based on inverse contrast normalization. *Neuroimage*. Jul 15 2015;115:269-80. doi:10.1016/j.neuroimage.2015.03.050

4. Hagmann PJ, L. ; Maeder, P. ; Thiran, Jean-Philippe ; Wedeen, V. ; Meuli, R. Understanding Diffusion MR Imaging Techniques: From Scalar Diffusion-weighted Imaging to Diffusion Tensor Imaging and Beyond. *Radiographics*. 2006;26:205-S223.

5. Mandl RCW, Schnack HG, Zwiers MP, van der Schaaf A, Kahn RS, Hulshoff Pol HE. Functional Diffusion Tensor Imaging: Measuring Task-Related Fractional Anisotropy Changes in the Human Brain along White Matter Tracts. *PLOS ONE*. 2008;3(11):e3631. doi:10.1371/journal.pone.0003631

6. Friedrich P, Fraenz C, Schlüter C, et al. The Relationship Between Axon Density, Myelination, and Fractional Anisotropy in the Human Corpus Callosum. *Cereb Cortex*. Apr 14 2020;30(4):2042-2056. doi:10.1093/cercor/bhz221

7. Landman BA, Farrell JA, Jones CK, Smith SA, Prince JL, Mori S. Effects of diffusion weighting schemes on the reproducibility of DTI-derived fractional anisotropy, mean diffusivity, and principal eigenvector measurements at 1.5T. *Neuroimage*. Jul 15 2007;36(4):1123-38. doi:10.1016/j.neuroimage.2007.02.056

8. Stampfli P, Sommer S, Manoliu A, et al. Subtle white matter alterations in schizophrenia identified with a new measure of fiber density. *Sci Rep*. Mar 15 2019;9(1):4636. doi:10.1038/s41598-019-40070-2

9. Bedford P, Hauke DJ, Wang Z, et al. The effect of lysergic acid diethylamide (LSD) on whole-brain functional and effective connectivity. *Neuropsychopharmacology*. Jul 2023;48(8):1175-1183. doi:10.1038/s41386-023-01574-8

10. Koutsouleris N, Borgwardt S, Meisenzahl EM, Bottlender R, Moller HJ, Riecher-Rossler A. Disease prediction in the at-risk mental state for psychosis using neuroanatomical biomarkers: results from the FePsy study. *Schizophr Bull*. Nov 2012;38(6):1234-46. doi:10.1093/schbul/sbr145

11. Perez VB, Woods SW, Roach BJ, et al. Automatic auditory processing deficits in schizophrenia and clinical high-risk patients: forecasting psychosis risk with mismatch negativity. *Biol Psychiatry*. Mar 15 2014;75(6):459-69. doi:10.1016/j.biopsych.2013.07.038
